# Assessment of Demographic, Genetic, and Imaging Variables Associated With Brain Resilience and Cognitive Resilience to Pathological Tau in Patients With Alzheimer Disease

**DOI:** 10.1001/jamaneurol.2019.5154

**Published:** 2020-02-24

**Authors:** Rik Ossenkoppele, Chul Hyoung Lyoo, Jonas Jester-Broms, Carole H. Sudre, Hanna Cho, Young Hoon Ryu, Jae Yong Choi, Ruben Smith, Olof Strandberg, Sebastian Palmqvist, Joel Kramer, Adam L. Boxer, Maria L. Gorno-Tempini, Bruce L. Miller, Renaud La Joie, Gil D. Rabinovici, Oskar Hansson

**Affiliations:** 1 Lund University, Clinical Memory Research Unit, Lund, Sweden; 2 Alzheimer Center Amsterdam, Department of Neurology, Amsterdam Neuroscience, Amsterdam University Medical Center, Amsterdam, the Netherlands; 3 Gangnam Severance Hospital, Department of Neurology, Yonsei University College of Medicine, Seoul, South Korea; 4 King’s College London School of Biomedical Engineering and Imaging Sciences, London, United Kingdom; 5 Dementia Research Centre, Department of Neurodegenerative Disease, University College London Institute of Neurology, London, United Kingdom; 6 Centre for Medical Image Computing, Department of Medical Physics, University College London, London, United Kingdom; 7 Gangnam Severance Hospital, Department of Nuclear Medicine, Yonsei University College of Medicine, Seoul, South Korea; 8 Division of Applied RI, Korea Institute Radiological and Medical Sciences, Seoul, South Korea; 9 Memory and Aging Center, Department of Neurology, University of California, San Francisco; 10 Department of Radiology and Biomedical Imaging, University of California, San Francisco; 11 Molecular Biophysics and Integrated Bioimaging Division, Lawrence Berkeley National Laboratory, Berkeley, California; 12 Associate Editor, *JAMA Neurology*; 13 Memory Clinic, Skåne University Hospital, Malmö, Sweden

## Abstract

**Importance:**

Better understanding is needed of the degree to which individuals tolerate Alzheimer disease (AD)–like pathological tau with respect to brain structure (brain resilience) and cognition (cognitive resilience).

**Objective:**

To examine the demographic (age, sex, and educational level), genetic (*APOE*-ε4 status), and neuroimaging (white matter hyperintensities and cortical thickness) factors associated with interindividual differences in brain and cognitive resilience to tau positron emission tomography (PET) load and to changes in global cognition over time.

**Design, Setting, an Participants:**

In this cross-sectional, longitudinal study, tau PET was performed from June 1, 2014, to November 30, 2017, and global cognition monitored for a mean [SD] interval of 2.0 [1.8] years at 3 dementia centers in South Korea, Sweden, and the United States. The study included amyloid-β–positive participants with mild cognitive impairment or AD dementia. Data analysis was performed from October 26, 2018, to December 11, 2019.

**Exposures:**

Standard dementia screening, cognitive testing, brain magnetic resonance imaging, amyloid-β PET and cerebrospinal fluid analysis, and flortaucipir (tau) labeled with fluor-18 (^18^F) PET.

**Main Outcomes and Measures:**

Separate linear regression models were performed between whole cortex [^18^F]flortaucipir uptake and cortical thickness, and standardized residuals were used to obtain a measure of brain resilience. The same procedure was performed for whole cortex [^18^F]flortaucipir uptake vs Mini-Mental State Examination (MMSE) as a measure of cognitive resilience. Bivariate and multivariable linear regression models were conducted with age, sex, educational level, *APOE*-ε4 status, white matter hyperintensity volumes, and cortical thickness as independent variables and brain and cognitive resilience measures as dependent variables. Linear mixed models were performed to examine whether changes in MMSE scores over time differed as a function of a combined brain and cognitive resilience variable.

**Results:**

A total of 260 participants (145 [55.8%] female; mean [SD] age, 69.2 [9.5] years; mean [SD] MMSE score, 21.9 [5.5]) were included in the study. In multivariable models, women (standardized β = −0.15, *P* = .02) and young patients (standardized β = −0.20, *P* = .006) had greater brain resilience to pathological tau. Higher educational level (standardized β = 0.23, *P* < .001) and global cortical thickness (standardized β = 0.23, *P* < .001) were associated with greater cognitive resilience to pathological tau. Linear mixed models indicated a significant interaction of brain resilience × cognitive resilience × time on MMSE (β [SE] = −0.235 [0.111], *P* = .03), with steepest slopes for individuals with both low brain and cognitive resilience.

**Conclusions and Relevance:**

Results of this study suggest that women and young patients with AD have relative preservation of brain structure when exposed to neocortical pathological tau. Interindividual differences in resilience to pathological tau may be important to disease progression because participants with both low brain and cognitive resilience had the most rapid cognitive decline over time.

## Key Points

**Question:**

Which demographic, genetic, and neuroimaging factors are associated with cognitive and brain resilience to pathological tau in patients with Alzheimer disease?

**Findings:**

In this multicenter, cross-sectional, longitudinal study of 260 cognitively impaired amyloid-β–positive participants, young age and female sex were associated with greater brain resilience, whereas higher educational level and cortical thickness were associated with greater cognitive resilience.

**Meaning:**

Cognitive and brain resilience may be associated with differential mechanisms, which may help explain interindividual differences in how well patients tolerate pathological tau.

## Teaser

This cross-sectional, longitudinal study of amyloid-β–positive individuals with mild cognitive impairment or Alzheimer disease dementia examines the factors associated with interindividual differences in brain and cognitive resilience to tau positron emission tomography load and to changes in global cognition over time.

## Introduction

Positron emission tomography (PET), fluid biomarker, and neuropathological studies^1,2,3,4,5^ have consistently demonstrated an association between increased pathological tau and decreased cognitive function and brain atrophy across the Alzheimer disease (AD) spectrum. However, the human brain is characterized by remarkable interindividual differences in coping with pathological insults because comparable amounts of pathological burden can result in variable levels of cognitive impairment or neurodegeneration.^6^ The degree of structural and cognitive loss relative to the pathological burden defines ones resilience, which is considered to be an aggregate term for multiple reserve-related concepts, such as cognitive reserve,^7^ brain reserve,^8^ or brain maintenance.^9^ Resilience can be further divided into brain resilience (BR) (better or worse than expected structural properties of the brain based on the pathological burden) and cognitive resilience (CR) (higher or lower than expected cognitive performance based on the pathological burden).^10,11^ To date, it is largely unknown which factors contribute to resilience to pathological tau, whether this differs between CR and BR, and whether the level of resilience is associated with rates of longitudinal cognitive decline.

Understanding why some individuals are more resilient to pathological tau than others may provide information for the development of resilience-enhancing therapies and help refine the prognosis in individuals with AD. We therefore measured the total burden of insoluble tau aggregates using flortaucipir labeled with fluor-18 (^18^F) PET in amyloid-β–positive persons with mild cognitive impairment (MCI due to AD) or AD dementia. We then computed individual resilience scores based on the degree of cortical thickness (BR) or cognition (CR) relative to the total tau burden. Finally, we tested whether demographic (age, sex, and educational level), genetic (*APOE*-ε4), and imaging markers (cortical thickness and white matter hyperintensities [WMHs]) are associated with between-person variability in CR and BR to pathological tau.^12,13,14,15^ On the basis of an emerging literature highlighting female-specific risks for developing AD,^12,16,17,18,19^ we were particularly interested in potential sex differences in resilience to pathological tau.

## Methods

### Participants

This cross-sectional, longitudinal study included 260 patients from the Memory Disorder Clinic of Gangnam Severance Hospital (Seoul, South Korea), the Swedish BioFINDER study at Lund University (Lund, Sweden), and the University of California, San Francisco (UCSF) AD Research Center (San Francisco, California) who underwent [^18^F]flortaucipir PET from June 1, 2014, to November 30, 2017. All patients tested positive for amyloid-β by PET and/or cerebrospinal fluid analysis (details were reported previously^20^), 83 were clinically diagnosed with MCI (referred to as MCI due to AD),^21^ and 177 were diagnosed with AD dementia.^22^ All underwent medical history and neurologic examination, magnetic resonance imaging (MRI), and neuropsychological testing. Data analysis was performed from October 26, 2018, to December 11, 2019. Written informed consent was obtained from all participants, and local institutional review boards (UCSF, University of California, Berkeley, Lawrence Berkeley National Laboratory, Lund University, Skåne University Hospital, the Swedish Medical Products Agency, and Gangnam Severance Hospital) for human research approved the study. This study followed the Strengthening the Reporting of Observational Studies in Epidemiology (STROBE) reporting guideline.^23^

### Acquisition of PET and MRI Data

The PET images were acquired using a Biograph micro-CT PET/CT scanner (Siemens Medical Solutions) in the Memory Disorder Clinic of Gangnam Severance Hospital, Discovery 690 PET scanner (GE Medical Systems) in the BioFINDER study, and a Biograph 6 Truepoint PET/CT scanner (Siemens Medical Solutions) for UCSF patients. The PET data were locally reconstructed into 4- × 5-minute frames for the 80- to 100-minute interval after injection.^15,24,25^ The MRIs were acquired on a 3.0-T Discovery MR750 scanner (GE Medical Systems) in the Memory Disorder Clinic of Gangnam Severance Hospital, 3.0-T Tim Trio or Skyra scanner (Siemens Medical Solutions) in the BioFINDER study, and a 3.0-T Tim Trio or Prisma scanner (Siemens Medical Solutions) at UCSF.

### T1-Weighted MRI Processing

The MRI data were centrally processed (at Lund University) using previously reported procedures.^20^ In brief, cortical reconstruction and volumetric segmentation were performed with the FreeSurfer software, version 6.0 image analysis pipelines.^26^ The magnetization prepared–rapid gradient echo (MP-RAGE) images underwent correction for intensity homogeneity,^21^ removal of nonbrain tissue,^22^ and segmentation into gray matter and white matter with intensity gradient and connectivity among voxels.^24^ Cortical thickness was measured as the distance from the gray matter–white matter boundary to the corresponding pial surface.^25^ Reconstructed data sets were visually inspected for accuracy, and segmentation errors were corrected. Cortical thickness was determined across the whole cortex for the primary analyses and in frontal, temporal, parietal, and occipital regions of interest for secondary analyses (eTable 1 in the Supplement).

### [^18^F]Flortaucipir PET Processing

PET images were first resampled to obtain the same image size (128 × 128 × 63 matrix) and voxel dimensions (2.0 × 2.0 × 2.0 mm) across centers. Next, PET images were centrally processed (at Lund University) using previously reported procedures.^20^ [^18^F]Flortaucipir images were motion corrected using the Analysis of Functional NeuroImages (AFNI) 3dvolreg data set, time averaged, and rigidly coregistered to the skull-stripped MRI. Voxelwise standardized uptake value ratio (SUVR) images were created using inferior cerebellar gray matter as the reference region.^27^ FreeSurfer software, version 6.0 parcellation of the T1-weighted MRI scan was applied to the PET data transformed to individuals’ native T1 space to extract mean regional SUVRs. We calculated mean [^18^F]flortaucipir SUVR across the whole cortex for the primary analyses and in frontal, temporal, parietal, and occipital regions of interest for secondary analyses (eTable 1 in the Supplement).

### Fluid-Attenuated Inversion Recovery MRI Processing

T2-weighted fluid-attenuated inversion recovery (FLAIR) images were available for 259 of the 260 study participants. We estimated WMH volumes using a segmentation method described elsewhere.^28^ In brief, this method builds a bayesian probabilistic data model based on a gaussian mixture model with an evolving number of components. Because of distribution skewness, data were log transformed before statistical analysis.

### Cognitive Data

Across the 3 centers, Mini-Mental State Examination (MMSE) and comparable tests for delayed episodic memory and category fluency were administered. We used data from local cognitively normal individuals as reference to create *z* scores for delayed episodic memory and category fluency. Furthermore, retrospective and prospective longitudinal MMSE scores were used to model changes in global cognition over time. We acquired 664 data points from 246 patients; 182 had at least 2 time points, with a median of 3 (range, 2-8). The mean (SD) interval between the first and last MMSEs was 2.0 (1.8) years.

### Statistical Analysis

We performed (separate) linear regression models between whole-cortex [^18^F]flortaucipir uptake and cortical thickness (eFigure 1 in the Supplement) and used the standardized residuals as a measure of BR (ie, lower than expected cortical thickness based on [^18^F]flortaucipir SUVR reflects low BR).^29,30^ The same procedure was performed using whole-cortex [^18^F]flortaucipir uptake vs MMSE (CR_MMSE_) (eFigure 1 in the Supplement), delayed episodic memory recall (CR_MEMORY_), and category fluency (CR_FLUENCY_) scores to obtain measures of CR (ie, a lower than expected cognitive score based on [^18^F]flortaucipir SUVR reflects low CR). Next, bivariate and multivariable linear regression models were performed with age, sex, educational level (as tertiles within each center because of cohort differences), *APOE*-ε4 status, WMHs (adjusted for intracranial volume), and cortical thickness (surface area weighted; in CR models only) as independent variables and BR and CR measures as dependent variables. The WMH volumes were used as variables and not included in the BR measure because the presence and directionality of an association between pathological tau and WMH volume are not clear.^31^ In addition to the original 2-step approach (ie, obtainment of residuals from the correlation between [^18^F]flortaucipir and thickness or cognition followed by bivariable and multivariate regression models), we modeled all variables together. In this linear regression model (simultaneous model), thickness or cognition was the dependent variable with [^18^F]flortaucipir SUVR and all variables as independent variables. In addition, we grouped patients into BR and CR tertiles and performed bivariate and multivariable multinomial logistic regression models using the same set of variables for BR and CR_MMSE_. Furthermore, because sex was our primary variable of interest, we tested for interactions between sex and each of the other variables with BR and CR_MMSE_. In secondary analyses, we examined the regional specificity of the findings by repeating the main analysis but this time using [^18^F]flortaucipir uptake, cortical thickness, and WMH volumes within 4 regions of interest (ie, frontal, parietal, temporal, and occipital) as measures of BR and CR_MMSE_. Finally, we examined clinical progression using MMSE score as the outcome variable in linear mixed models, including continuous measures of CR and BR, time, CR × time, BR × time, and BR × CR × time, adjusting for age, sex, and educational level. The model contained random intercept and slopes. For visualization purposes, we created a 4-level CR-BR variable (high CR and BR, high CR and low BR, low CR and high BR, and low CR and BR). The significance level was set at 2-sided *P* < .05. We used R, version 3.5.1 (The R Project for Statistical Computing) for the statistical analyses.

## Results

### Participants

A total of 260 participants (145 [55.8%] female; mean [SD] age, 69.2 [9.5] years; mean [SD] MMSE score, 21.9 [5.5]) were included in the study. The characteristics of the participants are presented in Table 1 and eTable 2 in the Supplement. The mean (SD) whole cortex [^18^F]flortaucipir SUVR did not differ between women and men (1.57 [0.40] vs 1.49 [0.39]; *F* = 2.595; *P* = .11).

**Table 1.** Baseline Characteristics of the Study Participants^a^

Characteristic	Total Sample (N = 260)	Gangnam Hospital		BioFINDER Study		UCSF	
		MCI Due to AD (n = 40)	AD Dementia (n = 55)	MCI Due to AD (n = 28)	AD Dementia (n = 51)	MCI Due to AD (n = 15)	AD Dementia (n = 71)
Age, y	69.2 (9.5)	71.4 (8.7)	73.2 (9.5)	71.7 (9.4)	70.9 (8.3)	63.6 (8.5)	63.9 (8.5)
Female, No. (%)	145 (55.8)	22 (55.0)	43 (78.2)	10 (35.7)	23 (45.1)	8 (53.3)	39 (54.9)
Educational level, y	13.3 (4.9)	11.9 (4.6)	10.1 (5.6)	12.5 (3.5)	12.1 (3.7)	17.5 (3.3)	16.7 (2.9)
MMSE score	21.9 (5.5)	25.3 (3.1)	18.7 (5.3)	25.7 (2.9)	21.2 (5.1)	27.0 (3.3)	16.7 (2.9)
CDR, sum of boxes	4.3 (3.0)	1.9 (0.9)	5.1 (2.2)	1.9 (0.9)	6.9 (3.9)	2.25 (0.9)	4.6 (2.1)
Delayed recall, *z* score	−3.0 (1.6)	−2.4 (0.5)	−2.30 (0.93)	−2.3 (1.3)	−3.22 (1.19)^f^	−2.88 (2.56)	−4.2 (1.87)
Category fluency, *z* score	−1.7 (1.1)	−0.8 (1.1)	−1.56 (1.03)	−1.4 (0.8)	−1.92 (0.94)	−1.04 (1.16)	−2.32 (1.01)
*APOE-*ε4 positivity, No. (%)	134 (57.3)	19 (47.5)	27 (50.0)	21 (77.8)	31 (66.0)	4 (44.4)	32 (56.1)
Global [^18^F]flortaucipir SUVR	1.53 (0.39)	1.24 (0.18)	1.51 (0.35)	1.30 (0.29)	1.53 (0.37)	1.40 (0.28)	1.83 (0.83)
Global cortical thickness, mm	2.18 (0.12)	2.26 (0.08)	2.20 (0.08)	2.14 (0.13)	2.08 (0.14)	2.30 (0.08)	2.19 (0.08)
Global WMH volumes, log mm^3^	3.60 (0.47)	3.69 (0.45)	3.80 (0.38)	3.68 (0.51)	3.67 (0.47)	3.23 (0.40)	3.38 (0.45)
Brain resilience, *z* score	0 (1)	0.55 (0.64)	0.10 (0.66)	−0.50 (1.13)	−0.87 (1.16)	0.92 (0.64)	0.22 (0.73)
Cognitive resilience, *z* score							
MMSE	0 (1)	0.35 (0.61)	−0.65 (0.94)	0.50 (0.64)	−0.13 (0.97)	0.82 (0.69)	0.08 (1.11)
Memory	0 (1)	0.20 (0.33)	0.46 (0.58)	0.27 (0.87)	−0.15 (0.84)	0.04 (1.78)	−0.48 (1.26)
Fluency	0 (1)	0.51 (1.1)	0.06 (0.91)	−0.04 (0.66)	−0.28 (0.96)	0.44 (1.09)	−0.24 (0.99)

Abbreviations: AD, Alzheimer disease; MCI, mild cognitive impairment; MMSE, Mini-Mental State Examination; SUVR, standardized uptake value ratio; UCSF, University of California, San Francisco; WMH, white matter hyperintensity.

^a^ Data are presented as mean (SD) unless otherwise indicated. Differences in baseline characteristics between diagnostic groups (ie, MCI due to AD and AD dementia separately) across centers were assessed using analysis of variance with post hoc Bonferroni tests for continuous variables and χ^2^ and Kruskal-Wallis tests with post hoc Mann-Whitney tests for categorical or ordinal variables.

### Brain Resilience

**Figure 1.**
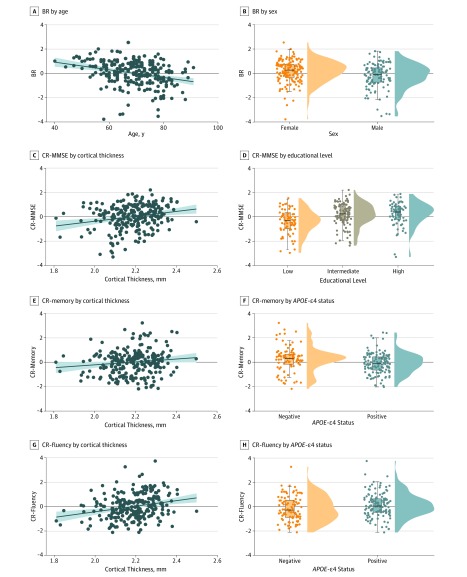
Key Associations of Brain Resilience (BR) and Cognitive Resilience (CR) With Cortical Thickness, Age, Sex, Educational Level, and *APOE*-ε4 Status MMSE indicates Mini-Mental State Examination.

**Figure 2.**
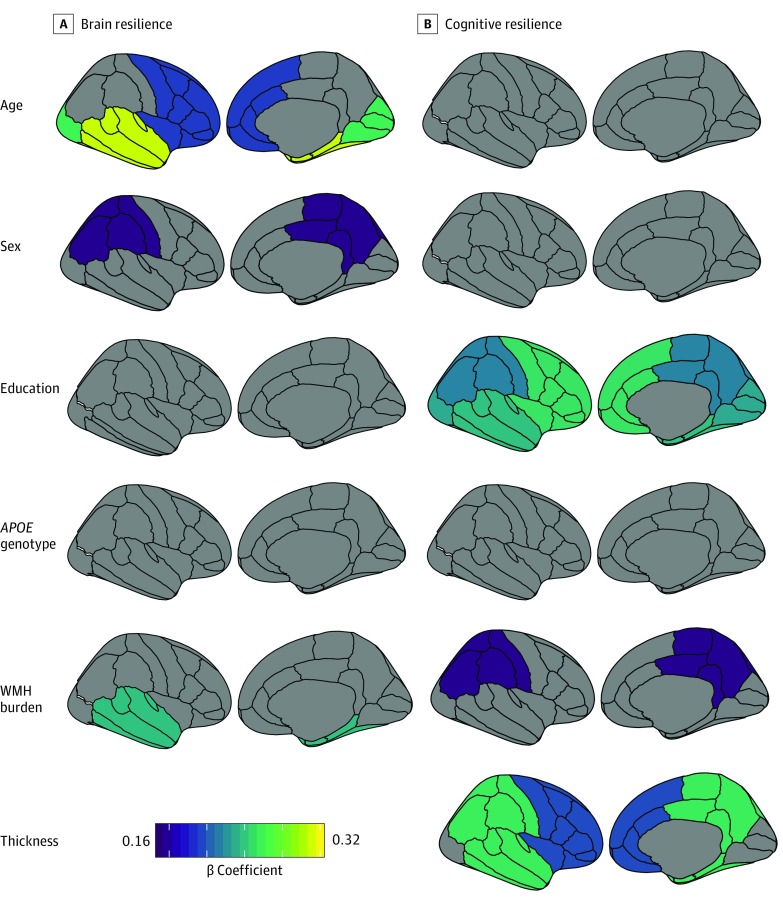
Regional Involvement of Key Factors Associated With Brain Resilience and Cognitive Resilience Significant β coefficients (*P* < .05 uncorrected for multiple comparisons) for the association between regional brain resilience (A) and cognitive resilience (B) and various indicators are plotted. Brain resilience is based on a linear regression between regional flortaucipir labeled with fluor-18 (^18^F) standardized uptake value ratio (SUVR) and regional cortical thickness in 4 regions of interest (ie, frontal, parietal, temporal, and occipital cortexes), whereas CR represents the residual of a linear regression between Mini-Mental State Examination scores and regional [^18^F]flortaucipir SUVR. WMH indicates white matter hyperintensity.

Bivariate models showed that female sex (standardized β [stβ] = −0.186; *P* = .003), younger age (stβ = −0.301; *P* < .001), and lower global WMH volumes (stβ = −0.282; *P* < .001) were associated with greater BR (Table 2 and Figure 1A and B). In the multivariable model, the associations with age (stβ = −0.202; *P* = .006) and sex (stβ = −0.147; *P* = .02) remained significant, but the association with WMH volumes did not (stβ = −0.140; *P* = .06). The simultaneous BR model yielded results comparable to those of the 2-step BR model (eTable 3 in the Supplement). In the 2-step and simultaneous BR models, the associations of age (stβ = −0.249; *P* = .001 in the 2-step model; stβ = −0.331; *P* < .001 in the simultaneous model) and sex (stβ = 0.125; *P* = .052 in the 2-step model; stβ = −0.139; *P* = .03 in the simultaneous model) with BR remained significant after additional adjustment for center. The multinomial logistic regression models were consistent with the linear regression approach (eTable 4 in the Supplement). No significant interactions were found between sex and any of the other variables on BR (eTable 5 in the Supplement). Regional analyses (ie, BR based on [^18^F]flortaucipir uptake vs cortical thickness within the 4 major lobes) showed that the associations between age and BR were present in frontal, temporal, and occipital cortexes but not the parietal cortex, whereas the association between sex and BR was only significant in the parietal cortex (Figure 2A). In addition, there was an association between WMH volumes and BR in the temporal cortex (stβ = −0.24; *P* < .001).

**Table 2.** Demographic, Genetic, and Imaging Variables Associated With Cognitive and Brain Resilience to Pathological Tau

Variable	Brain Resilience, Cortical Thickness		Cognitive Resilience					
			MMSE		Delayed Recall		Category Fluency	
	Standardized β	*P* Value	Standardized β	*P* Value	Standardized β	*P* Value	Standardized β	*P* Value
**Bivariate Models**								
Age	−0.301	<.001	−0.169	.008	0.060	.35	−0.052	.42
Sex	−0.186	.003	−0.12	.85	−0.077	.42	−0.064	.32
Educational level	−0.050	.43	0.258	<.001	−0.029	.65	0.118	.07
*APOE*-ε4 status	0.042	.53	0.061	.36	−0.182	.007	0.139	.04
Global WMH volume	−0.282	<.001	−0.244	<.001	0.061	.35	−0.131	.04
Global cortical thickness	NA	NA	0.241	<.001	0.115	.08	0.249	<.001
**Multivariable Models**								
No.	225	NA	225	NA	215	NA	216	NA
Age	−0.202	.006	−0.088	.23	0.027	.73	0.061	.42
Sex	−0.147	.02	0.055	.40	0.031	.65	−0.19	.78
Educational level	0.086	.61	0.232	<.001	−0.041	.56	0.098	.14
*APOE*-ε4 status	0.037	.56	0.022	.72	−0.170	.01	0.125	.06
Global WMH volume	−0.140	.06	−0.139	.06	0.056	.48	−0.078	.30
Global cortical thickness	NA	NA	0.233	<.001	0.155	.03	0.272	<.001

Abbreviations: MMSE, Mini-Mental State Examination; WMH, white matter hyperintensities.

### Cognitive Resilience

Bivariate models found that younger age (stβ = −0.169; *P* = .008), higher educational level (stβ = −0.258; *P* < .001), lower global WMH volumes (stβ = −0.244; *P* < .001), and greater global cortical thickness (stβ = 0.241; *P* < .001) were associated with greater CR_MMSE_ (Table 2). In the multivariable model, the associations with higher educational level (stβ = 0.232; *P* < .001) and cortical thickness (stβ = 0.233; *P* < .001) remained significant (Figure 1C and D), whereas the association with WMH volumes did not (stβ = −0.139; *P* = .06). The simultaneous CR_MMSE_ model yielded results comparable to those of the 2-step CR_MMSE_ model (eTable 3 in the Supplement). In both the 2-step and simultaneous BR models, the associations of global cortical thickness (stβ = 0.264; *P* < .001 in the 2-step model; stβ = 0.220; *P* < .001 in the simultaneous mode) and educational level (stβ = −0.255; *P* < .001 in the 2-step model; stβ = 0.233; *P* = .001 in the simultaneous model) with CR_MMSE_ remained significant after adjustment for center. The multinomial logistic regression CR_MMSE_ models were consistent with the linear regression approach (eTable 6 in the Supplement). We found an interaction between sex and WMH volumes on CR_MMSE_ (β [SE] = 0.571 [0.251]; *P* = .02), indicating that the associations between WMH volumes and CR_MMSE_ were more pronounced in women than in men. No interactions were found between sex and any of the other variables (eTable 5 in the Supplement).

Regional analyses (ie, CR based on MMSE vs [^18^F]flortaucipir uptake within the 4 major lobes) found that the associations between educational level and CR_MMSE_ were present across all regions of interest, whereas the associations between cortical thickness and CR_MMSE_ were present in the frontal, parietal, and temporal cortexes and those between WMH volumes and CR_MMSE_ in the occipital and parietal cortexes (Figure 2B).

For delayed episodic memory recall, *APOE*-ε4–negative participants had greater CR_MEMORY_ in both bivariate (stβ = −0.182) and multivariable (stβ = −0.170) models (Table 2 and Figure 1F). In addition, greater global cortical thickness (stβ = 0.155) was associated with greater CR_MEMORY_ in the multivariable model only (Table 2 and Figure 1 ). For category fluency, bivariate models indicated that *APOE*-ε4 positivity (stβ = 0.139), lower global WMH volumes (stβ = −0.131), and greater global cortical thickness (stβ = 0.249) were associated with greater CR_FLUENCY_, but only the association with global cortical thickness (stβ = 0.272) remained significant in the multivariable model (Table 2 and Figure 1G and H).

### Longitudinal Cognitive Decline

**Figure 3.**
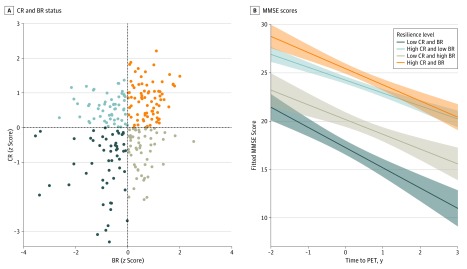
Longitudinal Cognitive Changes by Baseline Levels of Cognitive Resilience (CR) and Brain Resilience (BR) MMSE indicates Mini-Mental State Examination; PET, positron emission tomography.

A significant correlation was found between CR and BR (*r* = 0.245; *P* < .001) (Figure 3A). Figure 3B shows the estimated MMSE scores over time. The significant BR × CR × time interaction (β [SE] = −0.235 [0.111]; *P* = .03 for the 2-step approach;β = −0.378 [0.119]; *P* = .002 for the simultaneous model) indicates that the cognitive trajectories differed as a function of continuous BR and CR measures. For visualization purposes, we created a 4-level BR and CR measure. Individuals with low CR and BR had the steepest slope (annual β coefficient = −2.55; 95% CI, −2.95 to −2.14), followed by high CR and BR (annual β coefficient = −1.79; 95% CI, −2.43 to −1.14), high CR and low BR (annual β coefficient = −1.47; 95% CI, −1.79 to −1.15), and low CR and high BR (annual β coefficient = −1.47; 95% CI, −2.02 to −0.91) (Figure 3B and eFigure 3 in the Supplement).

## Discussion

In this multicenter study, we examined which demographic, genetic, and neuroimaging factors are associated with BR and CR against pathological tau as measured with [^18^F]flortaucipir PET. Results from this study suggest that women and young patients with AD have relative preservation of brain structure when exposed to neocortical pathological tau. Interindividual differences in resilience to pathological tau may be important with respect to disease progression because participants with negative BR and CR had the most rapid cognitive decline over time.

### Factors Associated With BR

The main finding of this study was the observation of greater BR in women compared with men even after adjusting for age, educational level, WMH volumes, and *APOE*-ε4 status. In other words, men had lower cortical thickness at similar levels of tau load. Under the assumption that tau aggregates cause neurodegeneration,^32^ this finding might suggest that female sex is protective against tau-induced cell death. Potential mechanisms include epigenetic changes, such as attenuated alterations in age-related gene expression that involves energy production and an upregulation of the immune system in women compared with men,^33,34^ as well as sex steroid hormone deficiencies that lead to sex-specific inflammatory responses to neuropathological insult.^35^ Our results are in line with a series of recent articles indicating that women compared with men had less cognitive impairment at similar levels of pathological tau,^18^ higher *APOE*-ε4–mediated cerebrospinal fluid phosphorylated tau levels,^17^ and greater pathological tau in the entorhinal cortex at similar levels of global amyloid-β burden in cognitively normal individuals.^12^ Although seemingly counterintuitive, these findings of higher resilience against tau may be congruent with epidemiologic observations of a higher prevalence of AD in women in the general population^36^ for at least 2 reasons. First, it is important to make the distinction between resistance and resilience against pathological tau.^10^ Our study found that women were possibly able to better preserve their brain structural properties after exposure to pathological tau, but that does not exclude the possibility that women are more prone to aggregate pathological tau than men. Second, our results fit with the higher life expectancy of women compared with men, especially given that advancing age is a major risk factor for the development of AD.^37^ Thus, although women may have a more favorable response to pathological tau, this benefit is counteracted by more years of high-risk exposure to pathological AD.

Our finding of greater BR in women could partially be associated with premorbid sex differences in cortical thickness, especially because we investigated brain structure (which is characterized by great interindividual variability in healthy brains) and not pathological molecular findings (which by definition are scarce in healthy brains) as the determinant of BR to pathological tau. Although some disparity exists in the literature, several studies^38,39,40,41^ have found greater thickness of especially the temporal and parietal cortexes in women than in men. Regional analysis of our data indicated that the association of sex with BR was most pronounced in the parietal cortex (Figure 2), but we found in a post hoc analysis that the main association of sex with [^18^F]flortaucipir uptake and cortical thickness was largely consistent across regions of interest (eFigure 2 in the Supplement). This finding suggests that the association between sex and BR is unlikely to be fully explained by premorbid sex differences in regional brain morphometry. However, longitudinal studies assessing actual change in cortical thickness are needed to confirm whether greater BR in women represents a baseline advantage, an attenuated rate of neurodegeneration compared with men, or a combination of both.

The other factor that contributes to BR was young age. This finding is in accordance with our a priori hypothesis because older patients are more likely to exhibit brain atrophy independent of tau burden (eg, owing to cerebrovascular disease, synaptic loss, or comorbid proteinopathies, such as transactive response DNA binding protein 43 kDa [TDP-43] or α-synuclein).^42,43^ Furthermore, neuronal repair mechanisms may become less efficient with age,^44^ which potentially increases the susceptibility to downstream effects of tau aggregates in older participants. Patients with early-onset AD, on the other hand, are characterized by greater baseline tau load and higher rates of tau accumulation rates compared with patients with late-onset AD.^15,45,46^

Although we did not find an association between educational level and BR, a previous PET study^47^ indicated that the association between pathological tau and glucose hypometabolism was mitigated by education. This finding could be explained by the use of structural (thickness) vs functional (hypometabolism) outcome measures^48^ or by differences in disease stage because education is possibly most beneficial in early clinical stages of AD.^49^ Furthermore, the effect sizes of BR and CR in the present study were small (range, 0.15-0.30),^50^ although only marginally smaller than those reported for treatment with acetylcholinesterase inhibitors—the current standard of care in MCI due to AD and early AD dementia—for cognitive (Cohen *d* = 0.29-0.51)^51,52^ and functional (Cohen *d* = 0.26)^52^ outcomes.

### Factors Associated With CR

Cognitive resilience was associated with the degree of cortical thickness and educational level, which is in line with previous studies reporting that education^13^ or highly correlated constructs, such as premorbid IQ,^53^ help to preserve cognitive function in patients with cortical pathological tau. Furthermore, the negative association of pathological tau with cognition was partially mediated by neurodegeneration.^54^ In bivariate models, age and WMH volumes were negatively associated with CR. The WMH volumes were also significant in the multivariable model (especially in occipitotemporal regions) (Figure 2), and their associations with CR_MMSE_ were most pronounced in women (eTable 5 in the Supplement). The association of WMH volumes with CR was potentially underestimated in this study because there were no overlapping neuropsychological tests across the 3 centers that specifically captured cognitive functions typically associated with cerebral small vessel disease, such as executive or attentional processes.^55^

### *APOE* Genotype

The *APOE* genotype was differentially associated with BR and CR. For CR, there was a remarkable dissociation because *APOE*-ε4 positivity was associated with lower CR based on memory performance, whereas absence of an *APOE*-ε4 allele was associated with lower CR based on a category fluency task. This finding aligns well with the literature because *APOE*-ε4 carriers have selective vulnerability of the medial temporal lobe and subsequent memory impairment, whereas *APOE*-ε4–negative patients with AD more often have cortical-predominant atrophy patterns in conjunction with nonamnestic cognitive deficits.^56,57,58,59^ Furthermore, we found no association between *APOE*-ε4 status and BR. Although *APOE*-ε4 positivity has been associated with a wide range of morphologic, hypometabolic, and functional alterations in cognitively normal persons,^60,61^ it is likely that in the clinically and biologically more advanced stage of disease in participants in the present study, neurodegenerative processes overwhelmed the more subtle premorbid association of *APOE*-ε4 with brain structure.

### Prognostic Value

We found an interaction between CR and BR and change in MMSE scores over time because individuals with low CR and BR progressed faster on the MMSE than individuals with low CR who had high BR. This finding suggests that CR and BR are not only associated with different demographic, genetic, and imaging features, they also provide distinct prognostic information.

### Strengths and Limitations

Strengths of the study include the relatively large sample of amyloid-β–positive individuals across the clinical spectrum of AD with imaging, genetic, and demographic data available. The study also has several limitations. First, there were only 3 equivalent cognitive tests available across centers. Although MMSE, delayed recall, and category fluency are important tests, several domains of cognition, such as executive functions or attention, were not sufficiently covered, and the CR scores were based on a single test. Second, educational level differed across the cohorts (mean [SD] years of education: 11 [5] in the Memory Disorder Clinic of Gangnam Severance Hospital, 12 [4] in the BioFINDER study, and 17 [3] in UCSF). This limitation was resolved by creating tertiles within each cohort, but we acknowledge that this approach potentially reduced the sensitivity to detect associations between educational level and BR and/or CR. Third, for the longitudinal analyses, we used an outcome measure (ie, MMSE) that was also used to determine CR. This approach was taken because there were no sufficient longitudinal data points available for the other cognitive tests (ie, delayed episodic memory and category fluency) or another global measure (eg, the clinical dementia rating scale). In addition, the MMSE is a crude measure to capture longitudinal changes in cognition. Fourth, some data were missing that could not be imputed because most relevant variables were already included in our statistical models. Fifth, amyloid-β pathologic findings were assessed using different modalities (PET and CSF analyses) and PET tracers; thus, a continuous measure of amyloid-β could not be entered as a variable in statistical analyses.

## Conclusions

In this study, female sex and young age were associated with greater BR against pathological tau, whereas higher educational level and cortical thickness were associated with greater CR. Furthermore, persons who had low CR and BR had the most rapid cognitive decline over time. Thus, CR and BR may be associated with differential mechanisms and may provide complementary prognostic information.

## Notes

Supplement. eTable 1. Region-of-interest (ROI) Definition eTable 2. Baseline Characteristics Stratified by Sex eTable 3. Multivariable Models Using Simultaneously Modeled Residual Scores eTable 4. Multinomial Logistic Regression Models for Brain Resilience (Thickness) eTable 5. Interactions Between Sex and the Other Predictors on BR and CR_MMSE_ eTable 6. Multinomial Logistic Regression Models for Cognitive Resilience (MMSE) eFigure 1. Associations Between FTP and MMSE and Regional Cortical Thickness eFigure 2. Main Effect of Sex on FTP vs Thickness Across ROIs eFigure 3. Change in Global Cognition by BR/CR Status When Modeled Simultaneously
