# Supplementary material for: Assessment of Demographic, Genetic, and Imaging Variables Associated With Brain Resilience and Cognitive Resilience to Pathological Tau in Patients With Alzheimer Disease
Source: JAMA Neurol. 2020 Feb 24;77(5):1–11. doi: 10.1001/jamaneurol.2019.5154 (PMC7042808; doi:10.1001/jamaneurol.2019.5154)
Supplement: Supplement. — eTable 1. Region-of-interest (ROI) Definition eTable 2. Baseline Characteristics Stratified by Sex eTable 3. Multivariable Models Using Simultaneously Modeled Residual Scores eTable 4. Multinomial Logistic Regression Models for Brain Resilience (Thickness) eTable 5. Interactions Between Sex and the Other Predictors on BR and CRMMSE eTable 6. Multinomial Logistic Regression Models for Cognitive Resilience (MMSE) eFigure 1. Associations Between FTP and MMSE and Regional Cortical Thickness eFigure 2. Main Effect of Sex on FTP vs Thickness Across ROIs eFigure 3. Change in Global Cognition by BR/CR Status When Modeled Simultaneously [file jamaneurol-77-632-s001.pdf]

Supplementary Online Content

Ossenkoppele R, Lyoo CH, Jester-Broms J, et al. Assessment of demographic, genetic, and imaging variables associated with brain resilience and cognitive resilience to pathological tau in patients with Alzheimer disease. *JAMA Neurol.* Published online February 24, 2020. doi:10.1001/jamaneurol.2019.5154

eTable 1. Region-of-interest (ROI) Definition

eTable 2. Baseline Characteristics Stratified by Sex

eTable 3. Multivariable Models Using Simultaneously Modeled Residual Scores

eTable 4. Multinomial Logistic Regression Models for Brain Resilience (Thickness)

eTable 5. Interactions Between Sex and the Other Predictors on BR and CR_{MMSE}

eTable 6. Multinomial Logistic Regression Models for Cognitive Resilience (MMSE)

eFigure 1. Associations Between FTP and MMSE and Regional Cortical Thickness

eFigure 2. Main Effect of Sex on FTP vs Thickness Across ROIs

eFigure 3. Change in Global Cognition by BR/CR Status When Modeled Simultaneously

This supplementary material has been provided by the authors to give readers additional information about their work.

© 2020 Ossenkoppele R et al. *JAMA Neurology.*

eTable 1. Region-of-interest definition

Combined Region	FreeSurfer Labels	FreeSurfer Label Names
Temporal	1001, 1009, 1006, 2006 1015, 1030, 1034, 2001, 2009, 2015, 2030, 2034	ctx-lh-bankssts, ctx-lh-inferiortemporal, ctx-lh-entorhinal, ctx-rh-entorhinal, ctx-lh-middletemporal, ctx-lh-superiortemporal, ctx-lh-transversetemporal, ctx-rh-bankssts, ctx-rh-inferiortemporal, ctx-rh-middletemporal, ctx-rh-superiortemporal, ctx-rh-transversetemporal
Parietal	1010, 1025, 2010, 2025, 1008, 1029, 1031, 2008, 2029, 2031	ctx-lh-isthmuscingulate, ctx-lh-precuneus, ctx-rh-isthmuscingulate, ctx-rh-precuneus, ctx-lh-inferiorparietal, ctx-lh-superiorparietal, ctx-lh-supramarginal, ctx-rh-inferiorparietal, ctx-rh-superiorparietal, ctx-rh-supramarginal
Occipital	1005, 1011, 1013, 1021, 2005, 2011, 2013, 2021	ctx-lh-cuneus, ctx-lh-lateraloccipital, ctx-lh-lingual, ctx-lh-pericalcarine, ctx-rh-cuneus, ctx-rh-lateraloccipital, ctx-rh-lingual, ctx-rh-pericalcarine
Frontal	1003, 1012, 1014, 1018, 1019, 1020, 1027, 1028, 1032, 2003, 2012, 2014, 2018, 2019, 2020, 2027, 2028, 2032	ctxcaudalmiddlefrontal, ctx-lh-lateralorbitofrontal, ctx-lh-medialorbitofrontal, ctx-lh-parsopercularis, ctx-lh-parsorbitalis, ctx-lh-parstriangularis, ctx-lh-rostralmiddlefrontal, ctx-lh-superiorfrontal, ctx-lh-frontalpole, ctx-rh-caudalmiddlefrontal, ctx-rh-lateralorbitofrontal, ctx-rh-medialorbitofrontal, ctx-rh-parsopercularis, ctx-rh-parsorbitalis, ctx-rh-parstriangularis, ctx-rh-rostralmiddlefrontal, ctx-rh-superiorfrontal, ctx-rh-frontalpole
Whole-cortex	1001, 1002, 1003, 1005, 1006, 1007, 1008, 1009, 1010, 1011, 1012, 1013, 1014, 1015, 1016, 1017, 1018, 1019, 1020, 1021, 1022, 1023, 1024, 1025, 1026, 1027, 1028, 1029, 1030, 1031, 1032, 1033, 1034, 1035, 2001, 2002, 2003, 2005, 2006, 2007, 2008, 2009, 2010, 2011, 2012, 2013, 2014, 2015, 2016, 2017, 2018, 2019, 2020, 2021, 2022, 2023, 2024, 2025, 2026, 2027, 2028, 2029, 2030, 2031, 2032, 2033, 2034, 2035	ctx-lh-bankssts, ctx-lh-caudalanteriorcingulate, ctx-lh-caudalmiddlefrontal, ctx-lh-cuneus, ctx-lh-entorhinal, ctx-lh-fusiform, ctx-lh-inferiorparietal, ctx-lh-inferiortemporal, ctx-lh-isthmuscingulate, ctx-lh-lateraloccipital, ctx-lh-lateralorbitofrontal, ctx-lh-lingual, ctx-lh-medialorbitofrontal, ctx-lh-middletemporal, ctx-lh-parahippocampal, ctx-lh-paracentral, ctx-lh-parsopercularis, ctx-lh-parsorbitalis, ctx-lh-parstriangularis, ctx-lh-pericalcarine, ctx-lh-postcentral, ctx-lh-posteriorcingulate, ctx-lh-precentral, ctx-lh-precuneus, ctx-lh-rostralanteriorcingulate, ctx-lh-rostralmiddlefrontal, ctx-lh-superiorfrontal, ctx-lh-superiorparietal, ctx-lh-superiortemporal, ctx-lh-supramarginal, ctx-lh-frontalpole, ctx-lh-temporalpole, ctx-lh-transversetemporal, ctx-lh-insula, ctx-rh-bankssts, ctx-rh-caudalanteriorcingulate, ctx-rh-caudalmiddlefrontal, ctx-rh-cuneus, ctx-rh-entorhinal, ctx-rh-fusiform, ctx-rh-inferiorparietal, ctx-rh-inferiortemporal, ctx-rh-isthmuscingulate, ctx-rh-lateraloccipital, ctx-rh-

© 2020 Ossenkoppele R et al. *JAMA Neurology.*

		lateralorbitofrontal, ctx-rh-lingual, ctx-rh-medialorbitofrontal, ctx-rh-middletemporal, ctx-rh-parahippocampal, ctx-rh-paracentral, ctx-rh-parsopercularis, ctx-rh-parsorbitalis, ctx-rh-parstriangularis, ctx-rh-pericalcarine, ctx-rh-postcentral, ctx-rh-posteriorcingulate, ctx-rh-precentral, ctx-rh-precuneus, ctx-rh-rostralanteriorcingulate, ctx-rh-rostralmiddlefrontal, ctx-rh-superiorfrontal, ctx-rh-superiorparietal, ctx-rh-superiortemporal, ctx-rh-supramarginal, ctx-rh-frontalpole, ctx-rh-temporalpole, ctx-rh-transversetemporal, ctx-rh-insula

© 2020 Ossenkoppele R et al. *JAMA Neurology.*

eTable 2. Baseline characteristics stratified by sex

	Total sample	Gangnam Hospital		BioFINDER study		UCSF	
		Females	Males	Females	Males	Females	Males
N	260	65	30	33	48	47	39
Age	69.2±9.5	72.9±9.4	71.5±8.7	69.3±9.1	72.6±8.1	62.2±8.5	65.7±8.1
Education (years)	13.3±4.9	9.8±5.1	13.1±5.0	11.4±2.9	12.9±4.0	17.0±2.6	16.7±3.4
MMSE	21.9±5.5	20.9±5.8	22.8±4.8	23.3±4.0	22.5±5.4	22.0±5.9	20.9±5.9
CDR, Sum-of-boxes	4.3±3.0	4.1±2.4	3.0±2.2	5.0±4.0	5.2±4.0	3.8±1.7	4.6±2.5
Delayed recall, z-score	-3.0±1.6	-2.3±0.8	-2.5±0.7	-2.7±1.5	-3.0±1.1	-4.2±1.9	-3.7±2.1
Category fluency, z-score	-1.7±1.1	-1.4±1.0	-0.9±1.3	-1.6±0.9	-1.9±0.9	-2.1±1.2	-2.1±1.1
APOE ε4 positivity, %	57.3	50.0	46.7	58.1	79.1	61.5	44.4
Global [^{18}F]Flortaucipir SUVR	1.53±0.39	1.40±0.31	1.38±0.34	1.50±0.38	1.41±0.34	1.84±0.37	1.66±0.42
Global Cortical thickness (mm)	2.18±0.12	2.23±0.09	2.22±0.08	2.12±0.14	2.09±0.13	2.22±0.08	2.20±0.10
Global WMH volumes (Log mm^3)	3.60±0.47	3.74±0.39	3.78±0.45	3.62±0.45	3.71±0.50	3.30±0.41	3.43±0.48
Brain resilience, z-score	0±1	0.32±0.72	0.24±0.62	-0.56±1.20	-0.86±1.1	0.46±0.69	0.20±0.83
Cognitive resilience MMSE, z-score	0±1	-0.35±0.98	0.02±0.85	0.26±0.74	-0.01±1.0	0.39±1.12	-0.05±1.01
Cognitive resilience Memory, z-score	0±1	0.39±0.54	0.25±0.41	0.15±1.00	-0.12±0.75	-0.47±1.34	-0.33±1.38
Cognitive resilience Fluency, z-score	0±1	0.12±0.90	0.54±1.18	0.04±0.88	-0.37±0.83	-0.02±0.99	-0.25±1.09

Baseline characteristics across centers stratified by sex.

© 2020 Ossenkoppele R et al. *JAMA Neurology.*

eTable 3. Multivariable models using simultanously modeled residual scores

	BRAIN RESILIENCE		COGNITIVE RESILIENCE	
	**Cortical thickness**		**MMSE**	
	Std. β	P	Std. β	P
Age	**-0.314**	**<0.001**	-0.104	0.178
Sex	**-0.155**	**0.012**	0.049	0.410
Education	-0.043	0.490	**0.217**	**<0.001**
APOE ε4 status	0.033	0.585	0.024	0.675
Global WMH volume	**-0.141**	**0.046**	-0.125	0.062
Global cortical thickness			**0.202**	**0.002**

© 2020 Ossenkoppele R et al. *JAMA Neurology.*

eTable 4. Multinomial logistic regression models for brain resilience (based on whole cortex [^{18}F]flortaucipir SUVR vs cortical thickness)

	OR (95% CI) Intermediate vs Low	P	OR (95% CI) High vs Low	P
**BIVARIATE:**				
**Age**	**0.959 (0.925-0.994)**	**0.021**	**0.905 (0.872-0.940)**	**<0.001**
**Sex**	0.635 (0.346-1.166)	0.143	**0.370 (0.198-0.691)**	**0.002**
**Education**	**0.648 (0.429-0.980)**	**0.040**	0.733 (0.485-1.109)	0.142
APOE ε4 status	0.967 (0.504-1.854)	0.920	0.995 (0.518-1.1913)	0.989
**WMH**	0.981 (0.947-1.107)	0.298	**0.916 (0.871-0.964)**	**0.001**
**MULTIVARIABLE:**				
**Age**	0.956 (0.918-0.997)	0.036	**0.922 (0.882-0.963)**	**<0.001**
**Sex**	0.876 (0.436-1.761)	0.710	**0.316 (0.146-0.684)**	**0.003**
Education	0.712 (0.451-1.124)	0.145	0.941 (0.575-1.540)	0.808
APOE ε4 status	0.987 (0.497-1.961)	0.971	0.999 (0.477-2.094)	0.999
WMH	1.001 (0.962-1.042)	0.948	0.972 (0.922-1.025)	0.290

© 2020 Ossenkoppele R et al. *JAMA Neurology.*

eTable 5. Interactions between sex and the other predictors on BR and CR_{MMSE}

	**BRAIN RESILIENCE**		**COGNITIVE RESILIENCE**	
Sex *	**Cortical thickness**		**MMSE**	
	β±SE	P	β±SE	P
Age	-0.013±0.015	0.398	0.016±0.014	0.229
Education	0.158±0.173	0.362	0.120±0.161	0.458
APOE ε4 status	-0.406±0.264	0.124	0.200±0.246	0.416
Global WMH volume	-0.013±0.275	0.963	**0.571±0.251**	**0.023**
Global cortical thickness			-0.420±1.041	0.687

© 2020 Ossenkoppele R et al. *JAMA Neurology.*

eTable 6. Multinomial logistic regression models for cognitive resilience (based on whole cortex [^{18}F]flortaucipir SUVR vs MMSE)

	**OR (95% CI) Intermediate vs Low**	**P**	**OR (95% CI) High vs Low**	**P**
**BIVARIATE:**				
**Age**	0.991 (0.958-1.025)	0.605	**0.948 (0.916-0.981)**	**0.002**
Sex	1.104 (0.596-2.042)	0.754	0.952 (0.513-1.765)	0.875
**Education**	**1.574 (1.028-2.410)**	**0.037**	**2.245 (1.456-3.463)**	**<0.001**
APOE ε4 status	0.937 (0.492-1.784)	0.937	1.272 (0.654-2.475)	0.479
WMH	0.998 (0.963-1.036)	0.930	0.964 (0.923-1.006)	0.095
**Cortical thickness**	7.118 (0.494-102.5)	0.149	**33.772 (2.055-554.9)**	**0.014**
**MULTIVARIABLE:**				
**Age**	0.988 (0.950-1.028)	0.565	**0.945 (0.90-0.986)**	**0.009**
Sex	1.136 (0.553-2.334)	0.729	1.367 (0.673-2.935)	0.422
**Education**	1.540 (0.970-2.446)	0.067	**2.068 (1.252-3.414)**	**0.005**
APOE ε4 status	0.862 (0.438-1.695)	0.667	1.197 (0.575-2.492)	0.631
WMH	1.013 (0.970-1.058)	0.562	1.002 (0.945-1.052)	0.933
**Atrophy**	3.808 (0.201-72.246)	0.373	**46.568 (1.592-1361.9)**	**0.026**

© 2020 Ossenkoppele R et al. *JAMA Neurology.*

eFigure 1. Associations between FTP and MMSE (left) and regional cortical thickness (right)

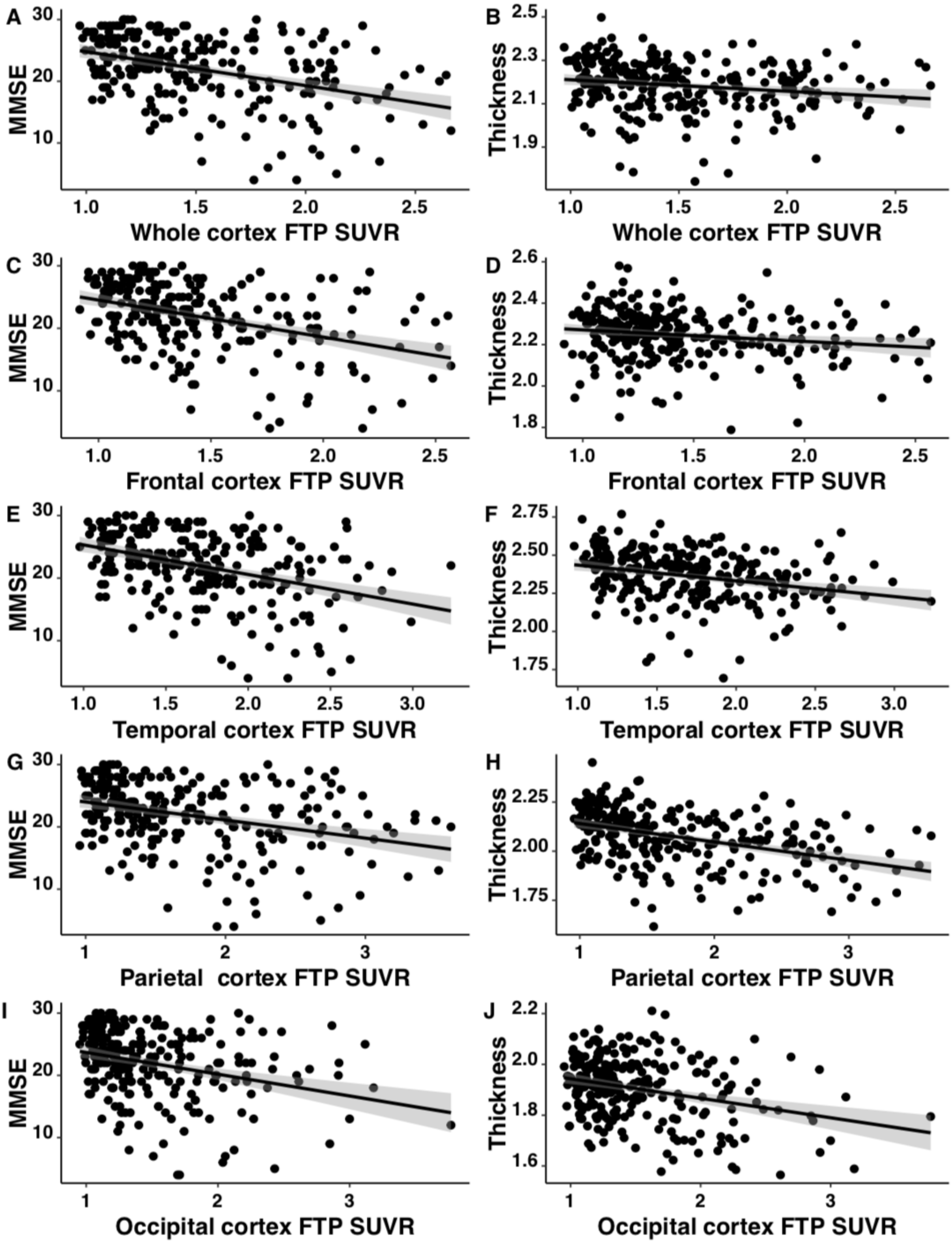

© 2020 Ossenkoppele R et al. *JAMA Neurology.*

eFigure 2. The main effect of sex on the relationship between [18F]Flortaucipir uptake and thickness across regions-of-interest

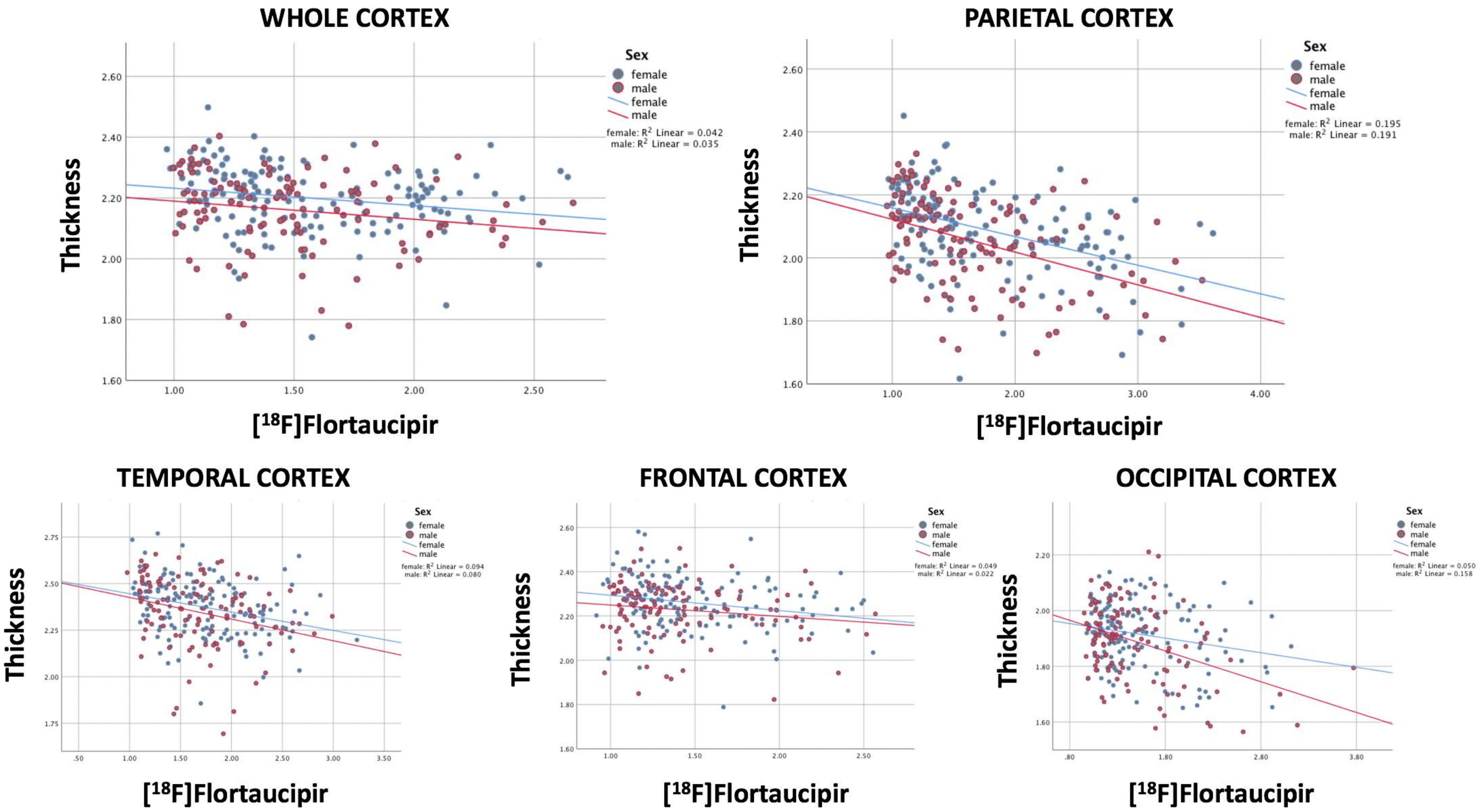

© 2020 Ossenkoppele R et al. *JAMA Neurology.*

eFigure 3. Change in global cognition as a function of BR/CR status modeled simultaneously

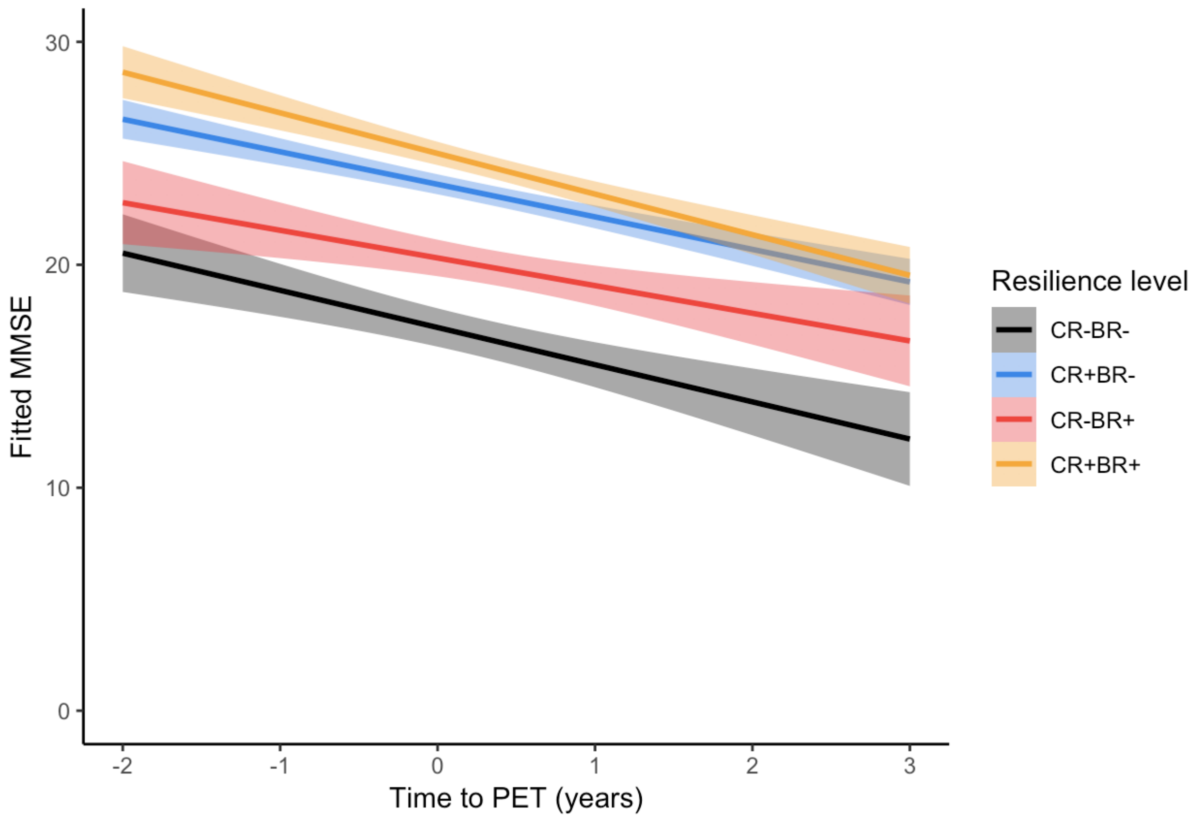

© 2020 Ossenkoppele R et al. *JAMA Neurology.*
